# Exploration the role of a clinical supervisor to improve the professional skills of medical students: a content analysis study

**DOI:** 10.1186/s12909-022-03473-w

**Published:** 2022-05-23

**Authors:** Mohammad Hasan Keshavarzi, Salimeh khalili Azandehi, Hamid Reza Koohestani, Hamid Reza Baradaran, Ali Asghar Hayat, Ali Asghar Ghorbani

**Affiliations:** 1 Clinical Education Research Center, School of Medicine, Shiraz University of Medical Sciences, Shiraz, Iran; 2 Social Security Organization, Valiasr Regional Hospital, Education & Research Unit, Ghaemshahr, Iran; 3 Social Determinants of Health Research Center, Saveh University of Medical Sciences, Saveh, Iran; 4 Center for Educational Research in Medical Sciences (CERMS), Iran University of Medical Sciences, Tehran, Iran; 5 Department of Medical Records, School of Paramedical Sciences, Mazandaran University of Medical Sciences, Sari, Iran

**Keywords:** Clinical supervisor,, Professional skills,, Responsibilities & characteristics,, Medical students

## Abstract

**Background and purpose:**

Clinical supervision supports learners and paves the way for effective and efficient learning in clinical settings. This study aimed to explain the responsibilities of clinical supervisors in clinical education wards to improve the professional skills of medical students.

**Materials and methods:**

In this qualitative study, we used the conventional content analysis approach. The sample consisted of 16 faculty members of medical sciences and medical graduates of Iranian universities. Purposeful sampling and semi-structured interviews were used to collect data. The Graneheim and Lundman method (2004) analyzed the data.

**Results:**

From the analysis of interviews, 2 themes, 8 categories, and 18 subcategories were obtained. “Clinical supervisor responsibilities” as a theme includes the categories: “Creating motivation in learner”, “Learner’s need recognition”, “Performance evaluation”, “Creating learning opportunities”, and “Professional ethics education”. And, the sub-categories were: “Creating a supportive atmosphere”, “Task assignment”,“Understanding training needs”, “Understanding individual needs”, “Periodic evaluation”, “Proper feedback’, “Reduce work stress”, “Learner engagement’ , “Learning Facilitation”, “Attention to the patient’s treatment”, and “Ethical observance in relation to patients”.

As the second theme “Clinical supervisor characteristics” included the categories of: “Scientific competence”, “Leading role”, and “Ethical model”. Their sub-categories are clustered as: “Knowledge of educational concepts”, “Mastery of professional concepts”, “Effective communication skills”, “Understanding managerial concepts”, “High resilience”, “Career commitment “, and “social commitment” .

**Conclusions:**

The clinical supervisor will improve the professional skills of medical students, which will improve the quality of services provided, train efficient graduates, and provide a safe and relaxing environment that leads to patient satisfaction.

## Introduction

Medicine is perceived as one of the sacred and praiseworthy professions in every society. In all countries, comprehensive and appropriate in-service education for the empowerment and efficiency of healthcare workers has been on the top agenda of educational planners [1].

One of the characteristics of medical education is the need to learn practical and communication skills along with cognitive and theoretical domains [2]. Clinical education is a dynamic process in which students gradually gain experience by attending the patient’s bedside and prepare their minds to solve the patient’s problems by using the experiences and logical arguments gained [3–5]. Clinical education is one of the critical stages of medical education that plays a significant role in shaping the professional abilities of learners and is an essential part of physician training as responsible for maintaining and promoting public health [6].

Providing the highest quality of clinical education to medical students is crucial for the delivery and care of public health [7]. The inability of medical students to acquire clinical skills could be attributable to the country’s universities’ weak and insufficient educational capacity [6]. Therefore, clinical education plays a pivotal role in the formation of professional identity [8].

Gold Hummer conducted the first investigations into clinical supervision in the 1960s [9] which focused on the data collection process during supervisions [10]. In 1973, Cogan developed and supported the concept of clinical supervision, noting the importance of professional interactions between stakeholders to contribute to the professional advancement of clinical educators. He also introduced clinical supervision stages that focused on planning, observation, and feedback [11]. Supervision includes ensuring inclusive patient safety during clinical care, providing informal feedback, and providing critical input to primary education and continuing education programs as well as monitoring and progress [12].

Ensuring inclusive patient safety during clinical practices is essential for clinical supervision [13]. Adequate supervision is an activity that creates growth and authority in the supervised group, facilitates the work of supervision, and supports both the client and the supervisor [14]. Tracing the quality of educational supervision in Tehran hospitals in Iran, Razmjoo et al. claimed no effective clinical supervision in hospitals for residents [15]. Unfortunately, today professors spend little time on clinical training. Thirty-five years ago, 55% of teaching time was supported for clinical training, but according to current studies, this time has been reduced to less than 16% [16]. Studies in Iran show that most attending physicians are aware of the importance of clinical supervision, but due to overlapping educational and medical responsibilities, less attention has been paid to clinical supervision in teaching hospitals [15].

Despite the benefits of a clinical supervision program; however, this position is not yet well designed in Iranian teaching hospitals. Students would be confronted with new environments that they do not have enough familiarity and experience with, and countless questions would arise in their minds. Since the medical students will be present in the clinical environment following the completion of basic sciences and preclinical courses and will gain insights on how to interact with the patient and family and various professional skills, the clinical supervisor position will significantly impact knowledge transfer students.

Therefore, this study has tended to explain the duties and characteristics of clinical supervisors in clinical settings to strengthen the professional skills of medical students in Iranian universities of medical sciences.

## Materials and methods

A qualitative approach and a conventional content analysis were used to describe a phenomenon in this research. Qualitative research was incorporated to achieve participants’ inner world and discover the meanings formed in a cultural context [17]. Content analysis is a suitable way to obtain valid results from textual data, create new knowledge, insights, and facts, and provide the direction that all organizational efforts will follow [18].

### Inclusion and exclusion criteria

The initial samples consisted of medical university faculty members who had teaching responsibilities in the clinical environment and had experience as the director of medical education in the teaching hospital. Also, the criteria for selecting students were those who graduated and have a certificate in medical education courses. Exclusion criteria were the participants’ unwillingness to continue cooperation.

### Data collection

Semi-structured interviews were used to collect information. The research objectives were explained before the interview. A written informed consent form was given to the interviewees. The interviewees explicitly agreed to record the interview. The interviews were conducted on an individual basis. During the interview, a general question was asked first, and then, as the interview progressed, the questions were controlled in the direction of the research goal. Questions asked in the interview were: the interview started with general open-ended question “Please express your understanding of educational supervision in a clinical setting?”, Moreover, and to have deeper insight into the concept under study, according to the participants’ responses more detailed and follow-up questions were raised as follows: “What qualities do you think a clinical supervisor should have?”, “How can you be a good clinical supervisor?”, “ what are the roles and responsibilities of a clinical supervisor in a clinical setting with students’ attendance?”. Also, a series of in-depth questions were asked: “Can you explain more?” Talk about “,” “you mentioned ...., what do you mean?”. Also, the students were asked questions like: “Please describe your experience from the clinical course you went through”, “Describe your experience with the activities and roles of a clinical supervisor in hospital wards?”, “Based on your experience with the clinical environment, what characteristics can the clinical supervisor have to improve the quality of learning?” Each interview lasted 35 to 45 minutes and was conducted by the first author. Participants were selected by the purposive sampling method from among the informants who provided the most data about the study subject. The interview site was either hospital or university environment decided upon by close interaction of the researcher and participants. Sixteen people participated in the interviews (Table 1).

**Table 1:** Demographic characteristics of the interviewees

P	Field of Study	Academic Ranking	Gender	CME
1	General Surgery	Assit. Professor	Male	MSc in Medical Education
2	Anesthesiologist	Assoc. Professor	Male	CME credit in medical education
3	Cardiologist	Assit. Professor	Male	MSc in Medical Education
4	Restorative Medicine	Assit. Professor	Male	CME credit in medical education
5	Internist	Assoc. Professor	Male	MSc in Medical Education
6	Emergency Medicine	Assit. Professor	Male	MSc in Medical Education
7	Anesthesiologist	Assoc. Professor	Male	CME credit in medical education
8	General Surgery	Assoc. Professor	Male	MSc in Medical Education
9	ENT specialist	Assit. Professor	Male	CME credit in medical education
10	Bone and joint specialist	Assit. Professor	Male	MSc in Medical Education
11	Social medicine	Assoc. Professor	Male	MSc in Medical Education
12	Medical Student	Medical Graduate	Male	CME credit in medical education
13	Medical Student	Medical Graduate	Female	MSc student in Medical Education
14	Medical Resident	Medical Graduate	Male	CME credit in medical education
15	Medical Resident	Medical Graduate	Male	MSc student in Medical Education
16	Medical Resident	Medical Graduate	Female	CME credit in medical education

### Data analysis method

Graneheim and Lundman (2004) [19] approach was used in data analysis [19]. In this study, the audio file was carefully transcribed by the first author immediately after each interview. Manuscripts and notes were added to the written text. The authors then reviewed the content several times to find other relations to the data, attain data immersion, and better understand what the interviewees were saying.

The meaning units were then identified and coded. The codes were merged based on similarity and were clustered into several sub-categories. Next, the sub-categories formed main categories based on similarities and differences. Attempts were made to have the greatest homogeneity within the categories. Incorporating the categories, the themes or abstract ideas emerged. Attempts were made not to involve the researcher’s assumptions in the analysis process. An example of analysis process is shown in Table 2.

**Table 2:** An example of analysis process

Meaning units	Codes	Subcategory	Category	Theme
A clinical supervisor can be a good teacher. Must know students’ capabilities and limits. Must know how to interact with the student. Must use modern teaching methods. Has the necessary training in creating interaction, teaching, and motivating students. Must use an active and interactive learning strategy in their training”….Be familiar with various student evaluation methods and practice how to give feedback to the student.	Good teacher, know students’ capabilities and limits, interact with the student, motivate student, interactive learning, active learning, student evaluation, feedback	Knowledge of educational concepts	Scientific competence	Clinical supervisor characteristics
My supervisor taught me how to be a professional. How do I know the patient. How get a medical history. How interact with the patient. He taught me how to convey bad news to the patient. How to empathize and show respect and treat each person with compassion. Make eye contact that patient feel comfortable.”	Be a professional, get a medical history, convey bad news, empathize, show respect, eye contact, feel comfortable	Mastery of professional concepts		

### Data rigidity

We employed the four critical criteria developed by Lincoln and Guba, including credibility, transferability, dependability, and confirmability [20].

Regarding credibility, the researcher’s continuous presence in the field provided the researcher with a better understanding of the phenomenon. Allocating enough time to collect and analyze data helped the authors gain deep insight into the data. For transferability, interviews, coding, and analysis were recorded and reported in detail in all the research processes. The dependability was established using some techniques. The opinions of the research team and double-checking of the codes were an agreement between the extracted categories and sub-categories. Regarding confirmability, several interviews, extracted codes and categories were reviewed by two faculty members familiar with data analysis of qualitative research.

### Ethical considerations

In coordination with the relevant authorities and obtaining the consent and approval of the participants, the interview was conducted. Confidentiality was also observed.

### Findings

Out of 16 participants, 11 were faculty members, and the remaining were graduating medical students and residents. From the analysis of interviews, two themes, eight categories, and 18 subcategories were obtained after coding and comparing the codes based on similarities and differences (Table 3).

**Table 3:** The themes, category and sub-category of findings

Themes	category	sub-category
Clinical supervisor responsibilities	Creating motivation in learner	Creating a supportive atmosphere
		Task assignment
	Learner’s need recognition	Understanding training needs
		Understanding individual needs
	Performance evaluation	Periodic evaluation
		Proper feedback
	Creating learning opportunities	Reduce work stress
		Learning Facilitation
		Learner engagement
	Professional ethics education	Attention to the patient’s treatment
		Ethical observance in relation to patients
Clinical supervisor characteristics	Scientific competence	Knowledge of educational concepts
		Mastery of professional concepts
	Leading role	Effective communication skills
		Understanding managerial concepts
		High resilience
	Ethical model	Career commitment
		Social commitment

### Theme 1: clinical Supervisor’s responsibilities

The theme clinical supervisor’s responsibilities refers to the of participants’ perception of the clinical supervisor’s responsibilities and activities that support students and patients. This theme include five main categories “creating motivation in the learner”, “ learner’s need recognition”, “performance evaluation”, “Creating learning opportunities,” and “professional ethics education”.

#### Creating motivation in the learner

It refers to the reinforcing factor and the desire of learners to learn and provide more service in the clinical setting. Its subcategories are “creating a supportive atmosphere “ and “task assignment”.

##### Creating a supportive atmosphere

It refers to creating psychological and even legal support in the clinical environment, part of which is the supervisor’s responsibility. *“The treatment environment is stressful and anxious for both the patient and the therapist. This is where a supporter, an encourager and a word of hope can keep your engine running, or either,”*… *“If a cold word or a certain behavior stops your engine, this is where the presence of a supporter is felt in this situation,”* (*P 7).*

##### Task assignment

In the clinical setting, students’ duties and roles should be clearly defined; doing so they perform their duties properly like other medical staff. *“If I know what I have to do in a clinical setting, I feel good, unlike the situation where I am unaware about what I am expected to do, from history-taking and physical examination to filling a medical history form and many other confusing things,” (P15).*

#### Learner’s need recognition

The clinical supervisor has to recognize the educational needs of the learners. Its sub-categories are “Understanding educational needs”, “Understanding individual needs”

##### Understanding training needs

The clinical supervisor should be aware of the needs of the various levels of students present in the clinical setting, including stagers, interns, and residents. “When *I was an intern, some of the cases we visited were very complicated and incomprehensible to our level of medical knowledge, I had a bad feeling there. We did not know if we had difficulty in learning or not, they are certain “fellow and residents cases, and this boundary was not clear to us and there was no one available to answer the questions.” (P16).*

##### Understanding individual needs

The clinical supervisor should be aware of the different characteristics of learners and their age and gender requirements. “*Although I had a lot of energy and ideas, I swallowed my idea for fear of being humiliated…. nobody seemed to understand us. Everyone was used to the usual routine, “*(P 14). *Some professors only had a good relationship with a certain gender group and did not pay attention to us, they did not even understand our situation” (P 16).*

#### Performance evaluation

It includes comprehensive monitoring and evaluation during the training course in the clinical environment to ensure students’ attainment of educational goals (i.e. Knowledge, attitude and skills). Its subcategories are “periodic evaluation” and “ proper feedback”.

##### Periodic evaluation

It refers to conducting assessments during the study and in each clinical ward and educational environment. *“We have now designed software to record the attendance and activities daily. The issue of student evaluation is significant. And not that at the end of the semester, the instructor, who does not even remember the students’ names, gives a grade carelessly and on no basis” (P5).*

##### Proper feedback

Providing feedback to address deficiencies and shortcomings is valuable. However, this feedback should be provided thoughtfully and appropriately to the learner. “*I have been receiving feedback from students every three weeks since I became in charge of supervising the students in the ward. I ask them, what were the good wards and the bad ones? Write the positive and negative points. When 10 out of 60 students write that this ward does not have good training, there is something wrong with that particular ward. I get the feedback, talk to the Head and the faculty of that ward, and retry again two weeks later.”(P 8).*

#### Creating learning opportunities

The educational supervisor can promote educational activities by using measures such as providing educational equipment, creating a healthy and fear-free learning atmosphere. Its subcategories are “reduce work stress “, “learner engagement” and “learning facilitation”.

##### Reduce work stress

Creating a calm atmosphere is the basis for effective learning and teaching. “*During this time, I saw many restless students, especially during the busy period, due to the heavy workload, stressful work environment, insomnia and many other things. Sometimes, I talk to them about my old experiences and big problems I had before and that they are all over now…. they cheer up and feel energetic”, (P 2).*

##### Learner engagement

Giving responsibility and involving participants in group learning activities (e.g. rounds, journal clubs, etc.) is part of engaging students. *“Some instructors do not put students in a position of scientific evaluation at all. The round is one-sided. They do not ask the student for any clinical judgment,” (P 14).*

##### Learning Facilitation

It refers to providing educational materials and equipment to facilitate educational activities. *“Many of the hospitals we worked there had poor teaching facilities; they didn’t have a good library to study or to find the references we needed. There were old computers, we had to bring our own. However, in the hospital environment, and the educational supervisor can provide the students with facilities like an amphitheater, a conference room to hold a journal club” (P 5).*

#### Professional ethics education

“Professional ethics education” deals with another responsibility of the clinical supervisor related to the patient. In addition to the student, the clinical supervisor must pay sufficient attention to the patient. Its sub-categories are “Attention to patient treatment “,“Ethical observance about the patient”.

##### Attention to patient’s treatment

It refers to the clinical supervisor’s sense of responsibility and attention to the patient’s treatment process. “*The patient who comes to the hospital with a thousand hopes is terrified and emotionally involved. The educational supervisor should also pay attention to this issue. On the other hand, care should be taken not to sacrifice education for treatment.” (P 9).*

##### Ethical Observance in relation to patients

It refers to the rules and regulations regarding the observance of ethical issues and the patient’s human dignity in the clinical environment. *“Rarely, however, there were professors with a weak perception of ethics. They shouted at the patients. They insulted them. They did not follow the charter of the patient’s rights. The patient did not dare to squawk either because they were unaware of their rights” (P 12)*.

### Theme 2: clinical supervisor characteristics

The second theme refers to a clinical supervisor’s wide range of scientific, ethical, professional, and communication characteristics. These characteristics are effective in effectiveness of professional activities in the clinical setting. This theme has three main categories: “Scientific competence”, “Leading role” and “Ethical model”.

#### Scientific competence

“Scientific competence” refers to a clinical supervisor’s competence and knowledge of professional and specialized issues. The clinical supervisor should have high clinical knowledge and skills to be able to supervise the care, health and medical performance of students. Its subcategories are “knowledge of educational concepts” and “mastery of professional concepts”.

##### Knowledge of educational concepts

It refers to the clinical observer’s familiarity with related concepts in teaching and learning. “*As a clinical professor, I have to be a teacher other than a specialized instructor. I should love education and have a concern about my field of education. I have to take a medical education course and increase my training knowledge, needs assessment, evaluation and teaching methods “(P 3).*

##### Mastery of professional concepts

It refers to the knowledge and skills of the clinical supervisor in the specialized and professional field of medicine and clinical wards. “*The person who claims to teach the student must have even more knowledge in that field than other instructors ... The student must feel that the teacher is capable, has mastery, this way he/she can trust the teacher because he believes the instructor has sufficient expertise in the field” (P1).*

#### Leading role

“ leading role is one of the traits of the clinical supervisor who guides learners in the process of teaching in a clinical environment. It guides the talents and energies of students toward achieving educational aims. This role guarantees the quality of care and patient safety in the hospital. Its subcategories are “effective communication skills”, “understanding managerial concepts”, “high resilience”.

##### Effective communication skills

This sub-category is related to appropriate communication skills between the supervisor and the learner. “*There are many times when I wish I had more time to talk to the teacher, but the connection between us is cut off and that connection is not established between the teacher and the student, especially during the internship.” (P 14).*

##### Understanding managerial concepts

Awareness of the basic concepts of management, including planning, organizing and monitoring, is an integral part of working in organizational environments. “*Everywhere in the world, it has been proven that you have to know something about management science when you take a job despite your expertise. A clinical supervisor must know about management factors and must have experienced how to interact and do conflict management” (P 8).*

##### High resilience

This sub-category addresses the resilience of the clinical supervisor in the face of shortcomings, deficiencies and criticisms in the educational environment. *“During this time, I witnessed a professor clashing with a student. In many places, complaints were lodged inside the university or even in the judiciary. I did not expect that student, but I expected more sobriety and better patience from the professor” (P 6).*

#### Ethical model

The clinical supervisor in the clinical setting should be a role model for other students. The clinical supervisor can influence the attitudes, values and behaviors of students. She/he can inspire students. Its sub-categories are “career commitment “, “social commitment “.

##### Career Commitment

It refers to being a moral role model and a good reputation. *“The first day we entered the ward, the teacher came earlier than everyone else. All the students gathered around him. He was a stylish professor. After a short introduction, the teacher said… listen …. This is a holy place, the patient’s body is holy. He/she allows us to examine his/her body and learn something, he/she has been very kind to us. Don’t say it smells bad! …. doric. I did not expect the teacher to say such things” (P 5).*

#### 2–3 Social Commitment

It refers to the clinical supervisor’s sense of responsibility to the organization, the community, the patient, and the students.” *Our teacher used to bring us together every week and said that God had allowed us to serve human beings. Seize this opportunity. Do not miss a moment of serving the people” (P 12).*

## Discussion

This study aimed to explain the responsibilities of a clinical supervisor in different hospital wards. There was a significant attitude toward the position of the clinical supervisor by participants of the study. The role of the clinical supervisor in the medical and educational environment has been neglected. Participants also noted both the responsibilities and individual characteristics of the clinical supervisor and its role in improving the quality of education and patient and medical students’ satisfaction.

In this study, creating motivation in learners included two subcategories: creating a supportive atmosphere and assigning tasks to learners. Determining duties and responsibilities in students makes them more motivated to perform their responsibilities. In her research, Åsa Alsiö et al. found that supervising medical students in clinical activities and being clear about the expectations for each role was effective in enhancing learners’ motivation [21].

Students in the clinical environment need the support and guidance of the instructors to strengthen their confidence in work, their sense of hope for the future, and to perform their professional duties properly. The main task of the clinical instructor is to provide opportunities for practical experience and to assess the status of patients. The clinical instructor should be available to answer questions, provide advice and guidance to students, and friendly support the clinical supervision of students in various situations [22, 23]. Zahraei et al.’s research also showed that students’ interest and motivation are influential factors in clinical education [24]. In their study, Ismaili and Nazer reported that about 66% of their study participants expressed future career prospects as an influential factor in academic achievement [25].

Another category attained in the present research is to offer proper feedback to medical students. Providing feedback makes students become better aware of their performance and its improvement and promotion and try to eliminate shortcomings. Fassihi Harandi et al. in their study considered providing feedback to students during the clinical course as one of the highest acclaimed categories obtained in the quality assessment of clinical education [26].

Another category highlighted in the present research is the creation of learning opportunities among learners. The presence of an educational supervisor paves the way for the transfer of teacher experiences to the learner. The learner can easily ask the instructor about the questions and issues that he will face in the future. In a study, medical students learned skills related to dealing with the patient, clinical examinations, and general procedures in the hospital. They consider the amount of learning to depend on motivation, patient access, and quality of teaching [27].

The survey of Zamanzad et al. on satisfaction and evaluation of medical students, clerks, and externs regarding the quality of clinical education in central wards showed their low satisfaction. Lack of time management by instructors, roaming in the wards and wasting time were repeatedly pinpointed as causes of dissatisfaction [28].

A calm and stress-free environment in students’ learning was extracted from the attained categories. Stressful environments lead to academic problems and reduced learning. In a study, stressors during medical education on medical students have been studied. The results showed that high stress and anxiety levels during medical education might negatively affect students’ learning and clinical success [29, 30].

In this study, observance of professional ethics and attention to patient rights were gleaned as the responsibilities of the clinical supervisor. Participants believed that an educational supervisor’s responsibilities are not limited to learners but also include the patient and patients’ rights. Through direct instruction, the clinical supervisor must instruct the student about patients’ rights, although the positive and negative messages of medical ethics in implicitly conveyed to the learner through the hidden curricula.

Batahi and Asayesh showed that more than half of medical students had moderate or poor knowledge of patient rights [31]. However, in his investigation, Mossadegh Rad indicated that the level of physician’s awareness of patients’ rights is excellent [32]. As a matter of course, the high level of awareness among employed physicians compared to students can be due to their more experience in the treatment and dealing more with patient rights issues. Tomlinson’s study showed that clinical supervision is related to the quality and safety of patient care [13].

Regarding the theme of Personal characteristics of clinical observers, the categories of scientific competence, leading role, and ethical model were obtained. Scientific competence included mastery of the professional career and expertise in education concepts. In this study, many instructors had much information about the concepts of teaching and learning, and their demographic information revealed that all of them had a master’s degree in medical education or had taken courses related to education.

Darvishpour and Javadi-Pashak, in a study entitled “Good Clinical instructors from the perspective of nursing students extracted six main categories, including academic ability, clinical skills, communication skills, evaluation skills, personality, and educational management in the clinical setting [33].

In our study, two categories of “career commitment “and” social commitment “were obtained following the professional ethics model. Students observe, analyze, and evaluate professional ethics through instructors’ practical behavior and attitudes in the clinical setting and wards.

Undoubtedly, students gain knowledge through learning, directly or indirectly, by observing the behavior of professors and how to deal with the patient. To put it in another way, students repeat what the instructors do, not what they say [34]. However, Torabizadeh et al. pointed out that one of the reasons for the non-observance of patient etiquette in medical centers is the lack of knowledge of medical staff about ethical principles and their observance in the clinic [35].

In this study, the category of “leading role” was introduced as another characteristic of the clinical supervisor. This category includes “effective communication skills”, “high resilience” and “understanding managerial concepts”. The clinical supervisor will need good communication with instructors, students at different levels, and patients and their companions. A prerequisite for this relationship can improve communication effectively. Rahimi et al. reported communication ability and individual personality as essential characteristics of a good instructor from students’ perspective [36].

In their review study entitled, “What makes a good clinical teacher in medicine?, Sutkin et al. reported the instructors’ knowledge, technical and clinical skills of clinical reasoning, positive communication with students in a supportive environment, communication skills and passion enthusiasm as the most common themes [37].

This study had some limitations. Small sample sizes for the student was the first limitation. We had to select the student who knew the concern of education and were familiar with the concept of education. Moreover, it was more time-consuming research. The final limitation was the lack of research in clinical supervision in Iran. However, the duties and position of clinical supervisor are already seen in nursing schools, and suitable information and research have been done in this area. It is suggested to design quantitative and qualitative works in this regard.

## Conclusions

In Iran, the clinical supervisor is the missing link in clinical wards and environments. Although some experienced professors in clinical departments offer this role based on intrinsic motivations and love of education, no special trustee has been designed for the clinical supervisor in the educational system. This has caused medical students to feel confused about their duties, responsibilities, and expectations, and they have a sense of wasting time and lack of support from instructors.

Improving the education of medical students in the clinical environment will enhance the quality of services and lead to the training of efficient and literate graduates and a safe and relaxing environment. In addition, it results in a sense of safety for patients and recipients of services in the clinical environment.

## Data Availability

The datasets generated and analyzed during the current study are not publicly available due to the ongoing analysis and research process but are available from the corresponding author on reasonable request.
